# Pastoral Care Provision for Medical Students: What Is the Role of the Clinical Teaching Fellow?

**DOI:** 10.1111/tct.70397

**Published:** 2026-03-17

**Authors:** Niamh T. McSwiney, Nicola Taylor, Steve Jennings

**Affiliations:** ^1^ University of Bristol Bristol UK; ^2^ Royal Bournemouth Hospital University Hospitals Dorset NHS Foundation Trust Bournemouth UK

## Abstract

**Background:**

The clinical teaching fellow (CTF) role is often taken by resident doctors between training programmes to develop skills within medical education. This project explored CTF experiences of pastoral care provision at one medical school in the United Kingdom (UK), specifically focusing on their own role and responsibilities to inform practical support strategies for doctors as future medical educators.

**Methods:**

This study adopted a qualitative, hermeneutic phenomenological approach. Five focus groups were conducted at different hospital sites where medical students undertake clinical placements, known individually as academies. The focus groups explored what pastoral support meant to CTFs and their experiences of providing pastoral support to medical students. Reflexive thematic analysis was undertaken collaboratively by the research team.

**Findings:**

The views of CTFs at different academies were generally aligned and four themes were identified. These included: (1) holistic pastoral care; (2) organisational influences: a mismatch of expectations; (3) challenges of the role; and (4) support for CTFs. The term pastoral care remains ill‐defined, but there was universal agreement amongst CTFs that it required a holistic approach to all student‐related issues. The near‐peer educational relationship between CTFs and medical students appears to be a fulfilling aspect of the role. Further clarity is required surrounding the expectations of the CTF role, which has led to indistinct boundaries surrounding the promotion of student well‐being.

**Conclusion:**

Future considerations include acknowledgment of the cognitive load associated with supporting students, defining CTF responsibilities within the current escalation pathways and providing preparatory and sustained training.

## 1 Background

As the number of medical schools in the UK rises, doctors and health care professionals are increasingly relied upon to provide teaching to medical students on clinical placements. Institutions are expanding the role of the clinical teaching fellow (CTF); typically, a qualified doctor who is interested in teaching.

The CTF role provides resident doctors with an out‐of‐programme experience, usually lasting 1 year, and many offer the opportunity to pursue postgraduate qualifications in medical education. The balance between teaching and clinical work varies between institutions, as well as in additional responsibilities, which may include supporting students' professional development, well‐being and offering mentorship through formal educational supervisor roles. Despite the increasing reliance on CTFs to teach medical students, there is a lack of primary research that offers a deeper understanding of their nonclinical roles, specifically pastoral support [1, 2, 3, 4]. Most literature regarding CTFs exists in the UK context. However, similar posts, for instance, educational fellows and teaching scholars, do exist internationally [5, 6, 7].

At one UK medical school, after 2 years of university‐based teaching, commonly referred to as preclinical years, medical students attend clinical placements at associated NHS hospitals, which, combined with an undergraduate education centre, are known as individual academies. The medical school employs a spiral‐type curriculum with early clinical exposure, in which core scientific knowledge and clinical skills are revisited with increasing complexity in later years. Clinical placements form the core of undergraduate training and medical students rotate between academies during the final 3 years of their degree, with a focus on case‐based learning, clinical skills and workplace‐based assessment. This method of attaching medical students with hospitals throughout the region is a practice replicated by other institutions throughout the UK and internationally [8].

Pastoral care is a commitment to the physical and emotional well‐being of others, commonly referred to in the context of students and teachers [9]. Despite familiarity with the phrase and an acknowledgement that academic roles in higher education involve pastoral care, it carries ambiguity, which can result in uncertainty for both students and faculty members [10, 11]. Furthermore, the literature suggests students in higher education are experiencing an increasing need for psychological support and are categorised as an at‐risk group for the development of mental health problems [12].

The aim of this study was to explore CTF experiences of pastoral care provision at their academies. Our research questions asked (Table 1):

**TABLE 1:** Research questions.

1 What are the personal experiences of CTFs in the support of medical students?
2 Are there any differences between the practice of pastoral care at individual academies?
3 What scope is there for a consensus vision of the pastoral role of the CTF?
4 How could the wider learning organisation better prepare and support CTFs for their role, specifically in relation to their pastoral capacity?

## 2 Methods

### 2.1 Study Design

A hermeneutic phenomenological approach aligned best with the research questions [13]. Five focus groups were conducted, with between four and seven participants based at individual academies. Focus groups were the most appropriate method, aligning to the study methodology, aims and questions, foregrounding shared and socially constructed experiences of the phenomena.

In April 2024, all CTFs currently employed were invited to participate by e‐mail, sent from an independent administrative mailbox, which included an electronic participant information sheet. No demographic variables were used within the sampling to maximise inclusion and foreground participation.

### 2.2 Study Setting

This study was conducted in a single medical school in the UK, which employs approximately 120 CTFs throughout seven academies.

### 2.3 Data Collection

**FIGURE 1:**
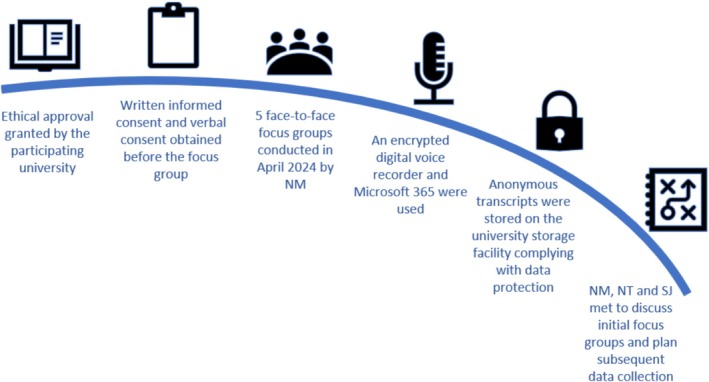
The data collection process.

Given the focus on establishing pastoral practices within and between academies, and the potentially sensitive nature of participants' experiences, the researchers organised CTFs working within the same academy to participate in separate focus groups to preserve the psychological safety of the participants and deepen collegiate discussion through shared experiences [14, 15] (Figure 1).

An indicative topic guide was used to balance participant‐driven narrative and key study concepts (Table 2).

**TABLE 2:** Topic guide for focus group discussion.

1 What does the phrase pastoral support mean to you?
2 When you first began this job how did you feel about providing pastoral support?
3 What are your experiences of providing pastoral support to medical students at your academy?
4 What are your thoughts on whether clinical teaching fellows should or should not be providing pastoral support?
5 If you think we should be providing pastoral support, what should it look like?
6 What are your thoughts on unifying the way each academy's clinical teaching fellows provide pastoral support?
7 Is there anything you think could help future clinical teaching fellows be prepared and supported to provide pastoral support?

### 2.4 Data Analysis

Reflexive thematic analysis was adopted [16]. N.M. and S.J. independently coded all five transcripts and discussed initial codes. The codes were agreed in June 2024. Themes and subthemes were critically discussed, adapted and refined by N.M., S.J. and N.T. between June and September 2024. Initial results were disseminated at the Association for the Study of Medical Education (ASME). There was conflicting guidance between the CTF induction, which advised signposting students to senior faculty and support services, and the practices followed at individual academies. Annual Scholarship Meeting 2024 and internally at the academy management meeting.

The authors were transparent about their positionality and integrated these insights into the discussion of codes, themes and subthemes. N.M. was interested in the professional development of the CTF role and at the time of the study was a CTF with experience of supporting students pastorally. S.J. is a teaching academic and N.T. is a clinician, both with experience of undergraduate and postgraduate pastoral support.

## 3 Findings

Participant characteristics are shown in Table 3.

**TABLE 3:** Participant characteristics in each focus group.

Characteristic	Focus group, *n* = 25 (%)
**Sex**	
Male	9 (36)
Female	16 (64)
**Number of completed years as a CTF**	
0 (currently in first CTF year)	19 (76)
1	5 (20)
2+	1 (4)
**Type of CTF role**	
Full‐time teaching	10 (40)
Part‐time teaching	15 (60)

**FIGURE 2:**
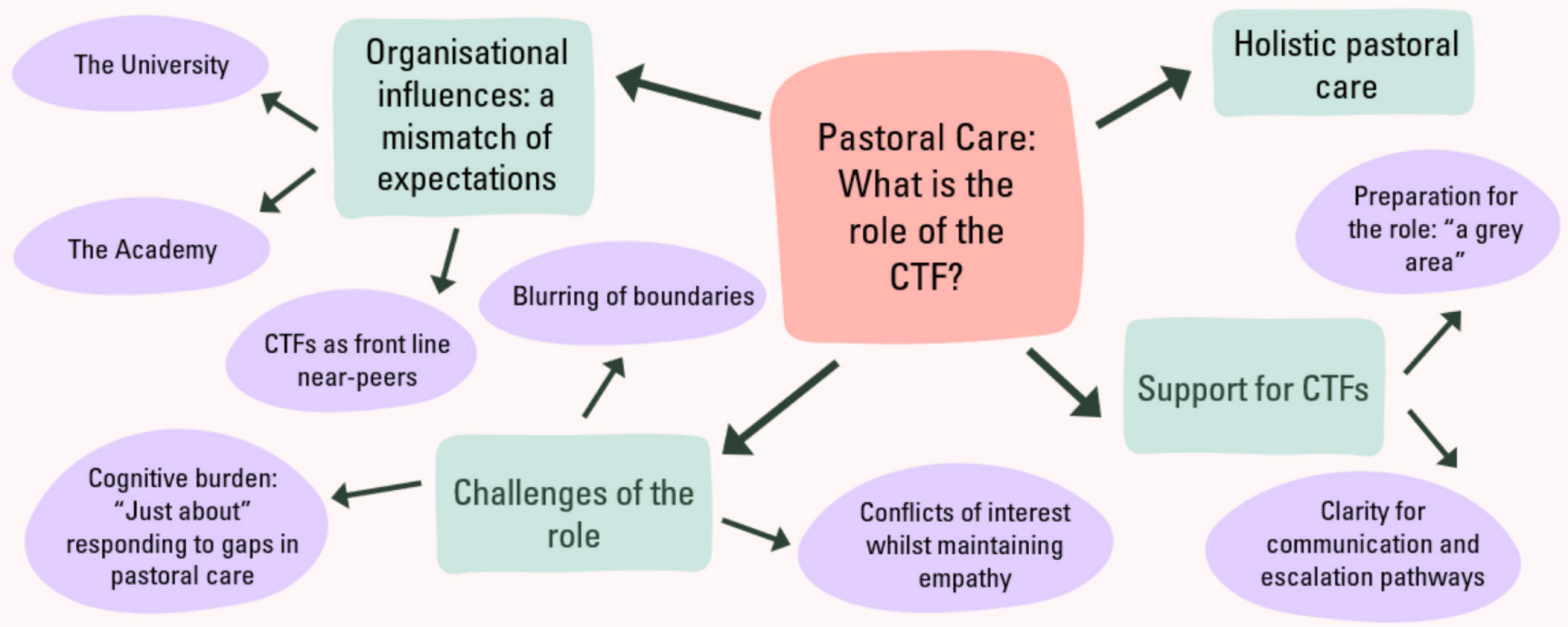
Themes (green) and subthemes (purple) emerging from the focus group discussions.

Four main themes were identified (Figure 2):

### 3.1 Holistic Pastoral Care

Most of the participants identified their role as involving pastoral care. Despite this, there were differences surrounding individual and group definitions. Initially, CTFs felt that pastoral care could be separated into academic and nonacademic related issues but that in practice these overlapped. There was unanimous agreement: pastoral care requires a holistic approach and could constitute anything a student was concerned about.

Pastoral care is recognising that students have personal lives and have families, have relationships and have a life outside of medicine and how that impacts how they're feeling day‐to‐day and their well‐being and mental health.

CTFs also acknowledged that whilst medical students were living on a hospital site, there was the potential for more pastoral problems to arise because of the separation between students and their usual support networks within the university‐based city.

I think pastoral support encompasses anything from how the student is feeling in terms of how they are getting on with their studies, but also how they're getting on living in the academy outside of (the university city), how they're getting on in their personal life, it's well‐being as a whole, related to both education, university and home life.

### 3.2 Organisational Influences: A Mismatch of Expectations

#### 3.2.1 The University

Participants considered there to be a geo‐cultural boundary between the central university and the surrounding academies. There was conflicting guidance between the CTF induction, which advised signposting students to senior faculty members and support services, and the practices followed at individual academies.

We feel so separate as academies to the central team, I don't know how (the CTF role) could be streamlined. Although it is only down the road, (the university faculty team) feel very far removed from what's happening on the ground sometimes

The message was very clear from the central induction, you are not (the student's) doctor, you are not their friend

When discussing the different support systems available to students, many CTFs were unsure of the different services or overwhelmed by the systemic complexity, unable to explain to the student the role of the service, their availability or the referral process. This included a lack of awareness from students and CTFs of the established role of personal tutors, a faculty member allocated to each medical student for their university career as a form of pastoral support.

I think the huge problem here is that there should be a dedicated senior who is their personal tutor centrally and they see ideally throughout their whole medical degree. That is the person who they should meet, even virtually. These complex issues that have been raised today are too much for us to handle

CTFs also felt that after referring a student to the university faculty team or student support service, there was little ongoing communication about that student. Whilst the support services manage student information with confidentiality, this led to CTFs feeling more removed from the university faculty and less able to support the students.

I don't think we're kept in the loop much from (the university). After that process of passing it off (we) don't really know what happens. I think you need to be because the students come back and like if you're concerned for maybe someone's safety

#### 3.2.2 The Academy

Participants considered academies to exert the most influence on CTF practice. This led to contrasting expectations on the pastoral role of the CTFs set by the university versus by the academy. For some CTFs, there was confusion around role boundaries, which were felt to be structural and maintained at an academy level, where they were asked to assess and triage student needs.

I think that goes back to the mismatch in expectation from the University and our academy. We did not have a (CTF) induction to the role from our academy but in the introduction talks to the students the message from the Deans was the CTFs door is always open to go to anytime of the day for support

The academies who provided the students with a designated figure known as a well‐being or pastoral lead, often a consultant clinician, was found to be a source of support by the CTFs.

I think the well‐being lead here is a great resource because we almost have immediate contact and response, and it feels so much more manageable than raising with the central team

#### 3.2.3 CTFs as Frontline Near‐Peers

For CTFs, student problems become more apparent with increased contact time and exposure to similar groups of students. CTFs felt students favoured face‐to‐face interaction and sought immediacy by contacting a CTF who was a front‐line presence at the academy and looked up to them as a role model.

We see our students over a prolonged period of time which has an impact on how you see pastoral care because you see them long enough for there to be issues within the group or for personal issues to become apparent

It is so hard to not have the pastoral side, you see the students on almost a daily basis for half a year. They look up to you as a role model

CTFs described a near‐peer type relationship with students, with pastoral support taking different approaches ranging from listening, reassuring, signposting and mentoring to having a more active role in problem‐solving. The conflicting information from different organisational levels resulted in the CTF's pastoral role being interpreted on a spectrum, with inconsistent approaches to students between academies and an unclear level of accepted responsibility for individuals.

I would love as someone who is that person in between the consultants and the students, that near‐peer, to help them develop as a professional to deal with things, motivate themselves, help them along

The data supported the idea that some CTFs self‐identify or identify other CTFs as being more interested or skilled in pastoral support, leading to boundaries with students becoming blurred into a therapeutic space.

Some people are better at doing pastoral activity at pastoral support and students warm to at times better and will approach more often potentially

### 3.3 Challenges of the Role

#### 3.3.1 Cognitive Burden: “Just About” Responding to Gaps in Pastoral Care

There was a common feeling of responsibility towards medical students' academic achievement. This was reflected in the idea that after building rapport with medical students, CTFs may appear to lack empathy when being unable to provide an immediate solution to a student's problem and a fear that signposting a student to resources wasn't doing enough in some individual cases.

We feel like (student problems are) our responsibility, it feels a bit like we are being dumped on a little, it's one thing to signpost but it's completely different to counsel or advice or mediate. Who do they go to if they can't access their personal tutor or GP or have to have a referral centrally? Do you know what I mean? It just seems like there is a gap here which is being just about filled by CTFs

In some academies where CTFs were also required to be educational supervisors, the balance between the role of providing support and signing academic competencies was a source of conflict and cognitive burden.

There is a huge mental burden. So a CTF was the person who had to tell the student they had failed and things had to be escalated to the university. But it was still the CTF doing all of that communication and the CTF who that student was upset to

There was concern amongst CTFs about personal repercussions associated with not supporting students enough or escalating appropriately. This resulted in careful and time‐consuming documentation after interactions with students for the purpose of record‐keeping.

I think there has to be a sharing of responsibility in terms of duty of care and legal responsibility because when it goes wrong. The last thing I want, something I think about a lot, is someone to go: Why didn't you tell anyone? Why didn't you escalate this to the university?

#### 3.3.2 Blurring of Boundaries

Ambiguity around the CTFs' role in relation to student problems made establishing and maintaining boundaries difficult. CTFs felt uncertain and unprepared about how to present a clear boundary to students, resulting in unequal levels of accepted responsibility for student well‐being and concern from some CTFs about progression of the role to managing students' mental health.

(Our role) is being kind of labelled as pastoral support, but actually we're managing their mental health. I think those are two distinct things, but they've become blurred now. I think (the students) expect you to provide pastoral support and I think that's because of the amount of time you spend with them as a teaching fellow. You're not seeing them once a week for a tutorial like a consultant is. You're so much more involved

#### 3.3.3 Conflicts of Interest Whilst Maintaining Empathy

CTFs felt discomfort in not doing more for students, reflecting the expectations from the different organisational levels, the influence of the near‐peer relationship and the difficulty in balancing their pastoral care role with other aspects of their job. They expressed a fear that through signposting students to support services that they may appear dismissive or unempathetic towards the individual problem, resulting in a disruption of the near‐peer relationship.

Then (a student) comes to you with their problems, they expect you to provide pastoral support, and then you've got the university saying no, don't do that, just redirect them to the services and it interrupts that relationship you've built up with them. That's the problem

### 3.4 Support for CTFs

#### 3.4.1 Preparation for the Role: “A Grey Area”

For CTFs, their awareness of the involvement of pastoral care in the role was reflected in the transparency of the recruitment process including the job description and the interview. Some participants also attended an additional local academy induction with input from previous CTFs, which were found to be particularly relevant.

When you start the job initially it's just figuring it out with a bit of trial and error. We have no formal training on providing pastoral care or how to deal with those issues

Often CTFs felt underprepared for the pastoral aspect of the role and prior to starting, some CTFs did not know what it meant in practice and were surprised at the volume of pastoral care issues, which arose. This left CTFs in a vulnerable position, reliant on a trial‐and‐error method when approaching student problems, with a conscious awareness of not being the students' doctor but relying on their clinical knowledge to stratify risk and concern.

I find it really tricky differentiating your role as a doctor versus your role as a teacher because at that point when you are risk assessing you're kind of thinking what's next and it's such a grey area

#### 3.4.2 Clarity for Communication and Escalation Pathways

CTFs often relied on the opinions of other CTFs to act as sounding boards for decisions regarding pastoral care. CTFs found sharing information surrounding a student problem a difficult balance of confidentiality, appropriate escalation and division of responsibility.

I feel the same hesitancy with sharing information with my colleagues. I do tell them things where I think it is relevant, but I haven't told the student I am going to. But that's for my colleagues to have an awareness of. Do I need to tell the student? I don't think so but I'm questioning now going into those conversations

CTFs felt that they required more support but were divided and unsure about what this would look like. There were positive responses from CTFs who had access to a well‐being lead and those who had a mental health first aid course as part of their local induction. CTFs felt inter‐academy communication about students requiring additional support through formal handovers may help avoid delay in the identification and implementation of support for students when moving between academies. There was a need for greater transparency surrounding the progress of a student who had been referred to the central, university‐based faculty team for support so that all teams locally and centrally were aware.

How can you spot the earliest signs (of a student problem)? What can you say to a (student) and then who are you going to sign posting to? I think the young person's (mental health first aid) course would have been more useful

## 4 Discussion

This research provides detailed insights into CTFs' experiences of providing pastoral care to medical students with a focus on how these experiences interrelate their role as both medical educators and clinicians.

Academics including CTFs are in frontline positions to be able to assist medical students facing problems [17]. Near‐peer teaching theory emphasises the benefits of an open teaching environment, which directly supports struggling students rather than necessitating a more formal escalation pathway [18]. In such an environment, near‐peer teachers provide information at an accessible level, encouraging a trusting relationship with an increasing interest in the student [19]. CTFs' comparatively high contact time provides a unique opportunity to build rapport and observe student progression, often cited as one of the most fulfilling aspects of the role [4, 20].

Academic faculty members should not be substitutes for healthcare professionals, but the medical background of CTFs adds a further level of complexity. Laws and Fielder [21] found an expectation that academic faculty provide pastoral care effectively despite not having the training, knowledge or preparation to undertake this additional position. Despite this, research has demonstrated that authority figures including teachers and supervisors have significant influence on the mental standing of students in their care, particularly through stimulation and positive reinforcement [10]. The potential influence of CTFs and their accessibility to students presents an ongoing challenge surrounding professional boundaries, with a need for training in pastoral care being identified by Ker et al. in 2018 [2].

With CTFs typically undertaking the role for 1 year, the development of a career merging medical education and clinical speciality training is largely uncertain [2, 3, 22]. Despite this, there are an increasing number of networks supporting novice medical educators [23, 24, 25]. Our data support the importance of embedding faculty development into CTF roles, a recognised method of enhancing existing teaching skills, to provide the relevant tools to deliver pastoral care and build a foundation for sustainable career progression [26]. Without signposting and support, the CTF role risks being perceived as a temporary teaching stop‐gap rather than a meaningful entry point into a career in medical education. To our knowledge, however, there is currently no research specifically addressing the faculty development of CTFs, representing an important gap in the literature.

### 4.1 Limitations

The participants sampled may have been more engaged in pastoral care debates than nonresponders. We endeavoured to ensure a representative spread of perspectives by engaging CTFs from the following five different academies; however, the sample remains regionally specific. Despite this, our analysis has revealed several emerging themes providing a novel and relevant insight into the experiences of CTFs, which offers a strong foundation for future research in the UK and internationally.

## 5 Conclusion

Clinical teaching fellows require ongoing professional development surrounding the nature of pastoral care problems amongst medical students, specifically mental health‐related. There is a need for prior preparation for the role, to better orientate CTFs with the support services and escalation pathways in place for medical students at different institutions. The workload must acknowledge the cognitive burden for CTFs and consider the importance of support for faculty members themselves. Defining CTF roles would allow for boundary setting, management of student expectations and effective balance of academic and nonacademic responsibilities.

## Author Contributions

**Niamh T. McSwiney:** conceptualization, investigation, methodology, project administration, writing – original draft, writing – review and editing. **Nicola Taylor:** conceptualization, supervision, writing – review and editing. **Steve Jennings:** conceptualization, methodology, resources, supervision, writing – review and editing.

## Funding

The authors have nothing to report.

## Ethics Statement

Ethical approval was granted by the University of Bristol Faculty Ethics Committee on 22 March 2024.

## Consent

Individual participant consent forms were signed prior to participation in the focus group.

## Conflicts of Interest

The authors declare no conflicts of interest.

## Data Availability

The data underlying this article cannot be shared publicly due to the privacy of the individuals who participated in the study.

## References

[1] D. Couchman , D. Donnachie , J. Tarr , and S. Bull , “Clinical Teaching Fellows, the New Norm?—Experiences of Fellows and Education Faculty,” Clinical Teacher 19, no. 4 (2022): 299–307. 35397149 10.1111/tct.13487 PMC9543777

[2] R. Ker , J. Guckian , and A. J. Bowey , “Just a Year Out’?–Challenges of the Clinical Teaching Fellow,” MedEdPublish 7 (2018): 239.

[3] I. M. Harris , H. McNeilly , D. J. Ward , A. J. Sitch , J. Parry , and S. Greenfield , “The Clinical Teaching Fellow Role: Exploring Expectations and Experiences,” BMC Medical Education 24, no. 1 (2024): 213. 38429703 10.1186/s12909-024-05207-6 PMC10908057

[4] I. M. Harris , N. Dennis , D. J. Ward , A. J. Sitch , J. Parry , and S. Greenfield , “Experiences as a Clinical Teaching Fellow: Interviews With Clinical Teaching Fellows in the West Midlands,” BMC Medical Education 24, no. 1 (2024): 1015. 39285404 10.1186/s12909-024-05958-2 PMC11406710

[5] C. van Heerden , W. Uahwatanasakul , B. Vaughan , and C. Delany , “Ripple Effect of a Clinical Teaching Fellow Programme in an Australian Paediatric Hospital,” Journal of Paediatrics and Child Health 56, no. 7 (2020): 1072–1076. 32100387 10.1111/jpc.14819

[6] L. D. Gruppen , D. Simpson , N. S. Searle , L. Robins , D. M. Irby , and P. B. Mullan , “Educational Fellowship Programs: Common Themes and Overarching Issues,” Academic Medicine 81, no. 11 (2006): 990–994. 17065863 10.1097/01.ACM.0000242572.60942.97

[7] Y. Steinert , L. Nasmith , P. J. McLeod , and L. Conochie , “A Teaching Scholars Program to Develop Leaders in Medical Education,” Academic Medicine 78, no. 2 (2003): 142–149. 12584092 10.1097/00001888-200302000-00008

[8] C. N. Nyoni , L. H.‐V. Dyk , and Y. Botma , “Clinical Placement Models for Undergraduate Health Professions Students: A Scoping Review,” BMC Medical Education 21 (2021): 1–26. 34863178 10.1186/s12909-021-03023-w PMC8642754

[9] M. Calvert , “From ‘Pastoral Care’ to ‘Care’: Meanings and Practices,” Pastoral Care in Education 27, no. 4 (2009): 267–277.

[10] R. J. Donovan , N. Henley , G. Jalleh , S. Silburn , S. Zubrick , and A. Williams , “The Impact on Mental Health in Others of Those in a Position of Authority: A Perspective of Parents, Teachers, Trainers and Supervisors,” Australian e‐Journal for the Advancement of Mental Health 5, no. 1 (2006): 60–66.

[11] D. Hughes and K. DuMont , “Using Focus Groups to Facilitate Culturally Anchored Research,” American Journal of Community Psychology 21, no. 6 (1993): 775–806. 10.1007/BF00942243 8085565

[12] F. Campbell , L. Blank , A. Cantrell , et al., “Factors That Influence Mental Health of University and College Students in the UK: A Systematic Review,” BMC Public Health 22, no. 1 (2022): 1778. 36123714 10.1186/s12889-022-13943-x PMC9484851

[13] B. E. Neubauer , C. T. Witkop , and L. Varpio , “How Phenomenology Can Help Us Learn From the Experiences of Others,” Perspectives on Medical Education 8 (2019): 90–97. 30953335 10.1007/s40037-019-0509-2 PMC6468135

[14] J. Kitzinger , “The Methodology of Focus Groups: The Importance of Interaction Between Research Participants,” Sociology of Health & Illness 16, no. 1 (1994): 103–121.

[15] J. Kitzinger , “Qualitative Research: Introducing Focus Groups,” BMJ 311, no. 7000 (1995): 299–302. 7633241 10.1136/bmj.311.7000.299 PMC2550365

[16] V. Clarke and V. Braun , “Thematic Analysis,” Journal of Positive Psychology 12, no. 3 (2017): 297–298.

[17] G. Hughes , M. Panjwani , P. Tulcidas , and N. C. Byrom , “Student Mental Health: The Role and Experiences of Academics,” (2018).

[18] H. G. Schmidt and J. H. C. Moust , “What Makes a Tutor Effective? A Structural Equations Modelling Approach to Learning in Problem‐Based Curricula,” (1995). 10.1097/00001888-199508000-00015 7646747

[19] E. R. Bowyer and S. C. K. Shaw , “Informal Near‐Peer Teaching in Medical Education: A Scoping Review,” Education and Health 34, no. 1 (2021): 29–33. 10.4103/efh.EfH_20_18 34213441

[20] G. Woodfield and M. O'Sullivan , “Clinical Teaching Fellows: Everyone's a Winner,” Clinical Teacher 11, no. 2 (2014): 136–140. 24629252 10.1111/tct.12084

[21] T. A. Laws and B. A. Fiedler , “Universities' Expectations of Pastoral Care: Trends, Stressors, Resource Gaps and Support Needs for Teaching Staff,” Nurse Education Today 32, no. 7 (2012): 796–802. 22633315 10.1016/j.nedt.2012.04.024

[22] S. Wilson , A. R. Denison , and H. McKenzie , “A Survey of Clinical Teaching Fellowships in UK Medical Schools,” Medical Education 42, no. 2 (2008): 170–175. 18230089 10.1111/j.1365-2923.2007.02933.x

[23] A. Chu , C. Morton , C. Pye , L. Ghani , and S. F. Smith , “Clinical teaching fellowships–enhancing the out of programme experience through a peer network,” Clinical Medicine 19, no. 3 (2019): 259. 31092525 10.7861/clinmedicine.19-3-259 PMC6542239

[24] D. Little , K. Butcher , S. Atkinson , D. Still , and J. Vasant , “A regional teaching fellow community of practice,” Clinical Teacher 11, no. 7 (2014): 516–519. 25417979 10.1111/tct.12229

[25] J. J. Lim , S. Birks , and C. Roberts , “How to … Navigate Specialised Programmes for Early‐Career Doctors in Medical Education,” Clinical Teacher 21, no. 6 (2024): e13832. 39489513 10.1111/tct.13832

[26] D. Simpson , K. Marcdante , and K. H. Souza , “The Power of Peers: Faculty Development for Medical Educators of the Future,” Journal of Graduate Medical Education 11, no. 5 (2019): 509–512. 31636817 10.4300/JGME-D-19-00613.1 PMC6795337

